# Mu Insertions Are Repaired by the Double-Strand Break Repair Pathway of *Escherichia coli*

**DOI:** 10.1371/journal.pgen.1002642

**Published:** 2012-04-12

**Authors:** Sooin Jang, Steven J. Sandler, Rasika M. Harshey

**Affiliations:** 1 Section of Molecular Genetics and Microbiology and Institute of Cellular and Molecular Biology, University of Texas at Austin, Austin, Texas, United States of America; 2 Department of Microbiology, Morill Science Center, University of Massachusetts at Amherst, Amherst, Massachusetts, United States of America; Uppsala University, Sweden

## Abstract

Mu is both a transposable element and a temperate bacteriophage. During lytic growth, it amplifies its genome by replicative transposition. During infection, it integrates into the *Escherichia coli* chromosome through a mechanism not requiring extensive DNA replication. In the latter pathway, the transposition intermediate is repaired by transposase-mediated resecting of the 5′ flaps attached to the ends of the incoming Mu genome, followed by filling the remaining 5 bp gaps at each end of the Mu insertion. It is widely assumed that the gaps are repaired by a gap-filling host polymerase. Using the *E. coli* Keio Collection to screen for mutants defective in recovery of stable Mu insertions, we show in this study that the gaps are repaired by the machinery responsible for the repair of double-strand breaks in *E. coli*—the replication restart proteins PriA-DnaT and homologous recombination proteins RecABC. We discuss alternate models for recombinational repair of the Mu gaps.

## Author Summary

Transposon activity shapes genome structure and evolution. The movement of these elements generates target site duplications as a result of staggered cuts in the target made initially by the transposase. For replicative transposons, the single-stranded gaps generated after the initial strand transfer event are filled by target-primed replication. However, the majority of known transposable elements transpose by a non-replicative mechanism. Despite a wealth of information available for the mechanism of transposase action, little is known about how the cell repairs gaps left in the wake of transposition of these majority elements. Phage Mu is unique in using both replicative and non-replicative modes of transposition. Our study finds that during its non-replicative pathway, the gaps created by Mu insertion are repaired by the primary machinery for double-strand break repair in *E. coli*, not by gap-filling polymerases as previously thought. This first report of specific host processes involved in repair of transposon insertions in bacteria is likely to have a broad significance, given also that double-strand break repair pathways have been implicated in repair of the retroviral and Line retroelement insertions.

## Introduction

**Figure 1:**
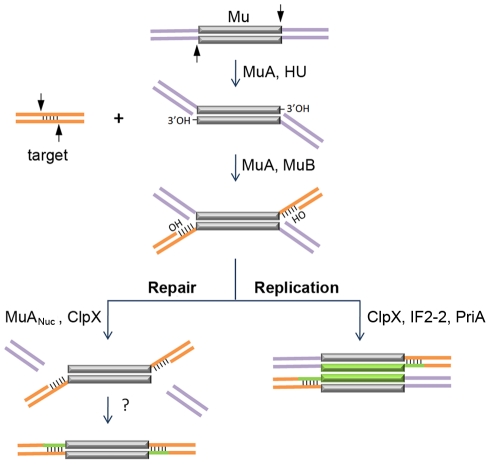
Known steps in replicative and non-replicative (repair) pathways of Mu transposition. The transposase MuA, in the presence of *E. coli* protein HU, first introduces single-stranded cleavages at the 3′ ends. With assistance from MuB, the 3′OHs at the cleaved ends are transferred by MuA to phosphodiester bonds spaced 5 bp apart in the target [3], [4]. The resultant branched strand transfer intermediate is processed alternately. During the lytic cycle, Mu is inserted in the chromosome, the target is also in the chromosome, so the purple flanking DNA is continuous with the orange target; transposition is intramolecular. The target OHs found in the strand transfer intermediate are used as primers to replicate Mu (green lines). ClpX, IF2-2 and other uncharacterized factors are required for disassembly of the transpososome followed by assembly of the PriA restart primosome on the Mu ends [5]. During integration of infecting Mu, the purple flanking DNA on the incoming Mu genome is non-covalently joined to itself via phage N protein; transposition into the chromosome target is intermolecular [9], [10], [11]. The branched strand transfer intermediate is resolved/repaired by MuA_Nuc_-mediated resection of the flap DNA [13], [14]. ClpX is required for this reaction. The remaining gaps are thought to be filled by host enzymes.

Transposable elements drive genome evolution in many ways – increasing DNA content, rearranging and mutating genes, as well as altering gene regulation [1]. Temperate phage Mu has played a pivotal role in our current understanding of how movable elements move [2]. A unique aspect of Mu is that, depending on the phase of its life cycle, it moves using either replicative or non-replicative modes of DNA transposition [3]. Most of our knowledge of Mu transposition is derived for the replicative pathway, where during lytic growth, Mu amplifies its genome by repeated transposition-replication events which exploit the host replication apparatus [4], [5]. *In vitro* experiments have established that in this pathway, the Mu transposase (MuA protein) mediates single-strand cleavages at Mu ends followed by strand transfer of the cleaved ends into target DNA; the latter reaction is greatly assisted by MuB protein (Figure 1). The resulting branched strand transfer joint is resolved by target-primed replication, which is initiated by the PriA primosome and completed by the Pol III holoenzyme, and results in duplication of the Mu genome after every round of integration. At the end of the lytic cycle, Mu genomes are packaged into phage heads such that they include host sequences (flaps) from both sides of a Mu insertion.

The non-replicative pathway of Mu transposition is only used when progeny phage infect new hosts [6], [7], [8]. Along with Mu DNA, the phage also inject into the host the phage N protein, which binds at the termini and converts the linear Mu genome into a non-covalently closed supercoiled circle [9], [10], [11]. Integration of the infecting Mu into the host genome follows the same initial nick-join steps of transposition established for the replicative mechanism *in vitro*; however, instead of target-primed Mu replication, the host flaps are resected and the gaps are repaired by unknown mechanisms [12] (Figure 1). Flap resection has not yet been demonstrated *in vitro*. This reaction is dependent *in vivo* on the cryptic endonuclease activity harbored within the C-terminal domain of the transposase MuA (designated MuA_Nuc_ in this study), as well as on the chaperone protein ClpX [13], [14]. ClpX is known to play an essential role during Mu replication, remodeling the Mu transpososome and enabling its transition to a replisome [5], [15] (Figure 1). The alternative choices for resolving the transposition intermediate, i.e. repair versus replication, must involve additional phage and host factors whose identity is not yet established.

The current study was undertaken to identify host factors involved in the repair of Mu insertions during the non-replicative infection pathway. To do so we used the Keio Collection, which is a set of 3,985 precisely defined, single-gene deletions of all nonessential genes in *Escherichia coli* K-12 [16], and screened for mutants defective in recovery of Mu::Cm insertions. Among the several mutants that gave a poor yield of Cm^R^ integrants, a majority of those that allowed Mu entry showed normal integration and replication of wild type Mu. By using two additional phage variants to re-screen/re-test in order to eliminate those defective in maintenance of a stable prophage state, we narrowed the search to a small subset of the mutants. Included among these were mutants in the homologous recombination pathway - *recA, recB, recC*. Two mutants - *priA* and *dnaT* – were defective in Mu replication as expected, but were unexpectedly defective in the recovery of insertions despite being proficient in Mu integration. The data show that Mu insertions are repaired by the replication restart machinery and homologous recombination proteins.

## Results

### *E. coli* Keio mutant screen with wild-type Mu

**Figure 2:**
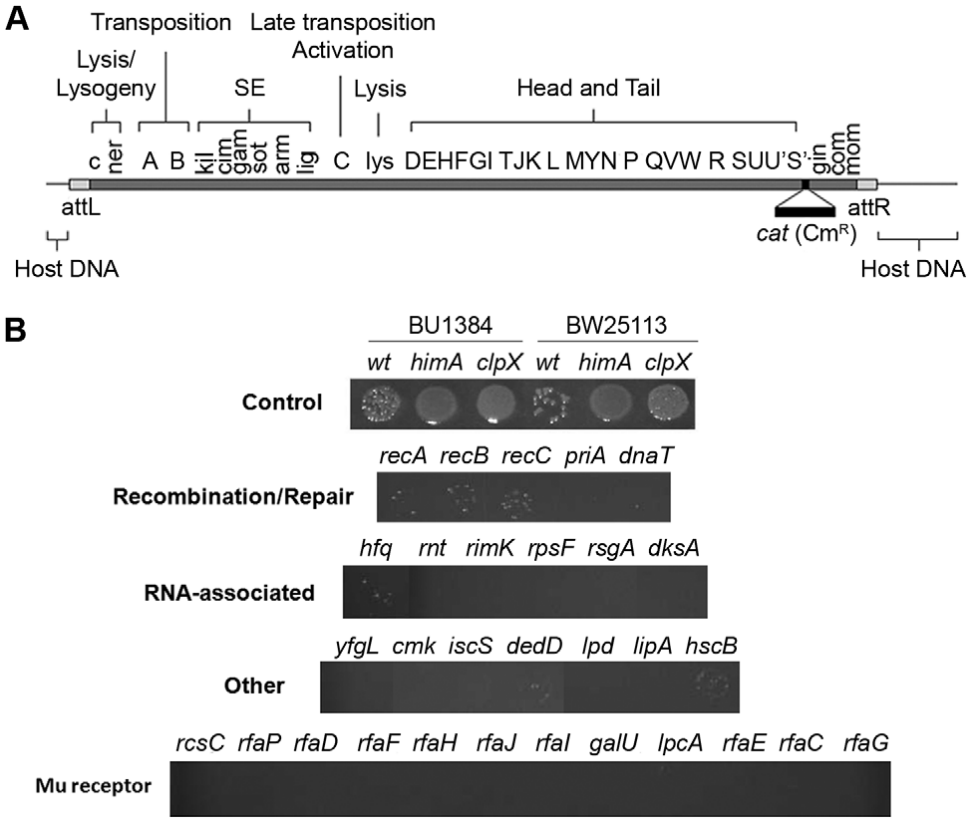
Identification of *E. coli* mutants in the Keio library defective in recovery of Mu::Cm insertions. (A) Functional map of the Mu genome packaged within a phage particle, showing position of inserted Cm^R^ cassette, and host or flap DNA attached to both ends. The SE (semi-essential) region contains 14 orfs [27]; only those assigned a phenotype/function are indicated [2]. (B) Cultures infected with Mu::Cm were spotted on Cm plates as described in Methods. Control panel: Expected results from infection of two different wild type and their derivative mutant strains - *clpX* and *himA* - that do not support Mu replication. Bottom four panels: Final set of mutants from the Keio screen showing lower Mu::Cm lysogen recovery compared to the wild type strain, grouped into indicated categories.

A functional map of the Mu genome is shown in Figure 2A. A ∼1 kb *cat* cassette encoding chloramphenicol (Cm) resistance was inserted into a non-essential region of the prophage genome (see Materials and Methods). Phage derived from this strain were used to infect the Keio mutant collection (see Table 1 for strain information), which occupies forty-eight 96-well plates, and spotted on agar slabs containing chloramphenicol to select for Mu lysogens as described in Methods. The control panel in Figure 2B shows results expected for known hosts where Mu integrates, but either does or does not replicate. In our standard wild type host BU1384 where Mu replicates, ∼90% of the infected cells undergo lytic growth and lysis, and ∼10% of the survivors (i.e. ∼1% of input cells) are lysogens. Mu fails to replicate in isogenic strains carrying either a *himA* or a *clpX* null mutant allele. *himA(ihfA)* codes for one of the two subunits of the regulatory protein IHF, which is required for early Mu gene transcription [2], [17], and ClpX is essential for Mu replication [5], [18]. Both of these mutant strains support Mu integration [12], [13], [19]. A larger number of Cm^R^ colonies are recovered in these strains compared to wild type because Mu does not undergo lytic development. Similar differences in the recovery of Cm^R^ colonies were seen in the wild type Keio strain BW25113 and its isogenic *himA* and *clpX* derivatives. In our screen for repair-defective mutants, we expected to identify mutant spots with either no Cm^R^ colonies or with fewer colonies than wild-type.

**Table 1:** Strains used in this study.

Strain	Genotype	Source (ref.)
***Mu prophage strain***		
MP1999	*recB recC sbcB malF*::Mu *cts62*	Martin Pato
BU1717	F' *pro lac*::Mu *cts62 Bam*1066 Su−	[22]
BU1091	F' *pro lac leu*::Mu *cts62* Amp	[19]
MH3491	Mu *cts62 Aam*1093 Su+	[23]
CW45	MP1999 with *cat* at 35040 nt of Mu	[13]
SJ17	BU1717 with *cat* at 35040 nt of Mu	This study
SJ18	MH3491 with *cat* at 35040 nt of Mu	This study
SJ19	BU1717 with Δ*SE::cat* in Mu	This study
***Plasmid***		
pJG4	9myc -MuB expressed from pET28a	Jun Ge
***Host strain***		
BU1384	F− Δ*pro lac* Su+	[19]
BU1382	BU1384, *himA*Δ*82*	[19]
CW11	BU1384, *clpX*::kan	[13]
BW25113	*rrnB*3 Δ*lacZ*4787 *hsdR*514 D(*araBAD*)567 Δ(*rhaBAD*)568 *rph*-1	Keio collection
SS996	Δ(*attB*)::p*sulA*-*gfp*	[35]
JC19328	Δ(*recA-srl*)306::Tn10	[34]
SS8872	Δ(*recB*)100::kan	Sandler Lab
SS8775	Δ(*recBCD*)::kan	Sandler Lab
SS1448	*priA*2::kan Δ(*attB*)::p*sulA-gfp*	[35]
SS1411	zji-202::Tn10 *dnaT*822 Δ(*attB*)::p*sulA-gfp*	[35]
SS1443	Δ(*priB*)302 Δ(*attB*)::p*sulA-gfp*	Sandler Lab
SS3403	*priC*303::kan Δ(*attB*)::*sulAp-gfp*	Sandler Lab
SS2357	Δ(*polA*)501::kan	Sandler Lab
SS3116	*priA*301 Δ(*attB*)::p*sulA-gfp*	Sandler Lab
SS1441	*priA*300 Δ(*attB*)::p*sulA-gfp*	[66]
SS2400	*dnaC*809,820 p*sulA-gfp* thr+	Sandler Lab
SS7087	*priA*2::kan *dnaC*809,820 Δ(*attB*)::p*sulA-gfp*	Sandler Lab
SS7086	zji-202::Tn10 *dnaC*809,820 *dnaT*822 Δ(*attB*)::p*sulA-gfp*	Sandler Lab
SS767	*malE*::Tn10 *lexA*3	[65]
SS749	Δ(*recA-srl*)306::Tn10 *priA*2::kan	Sandler Lab
SS768	*priA*2::kan *lexA*3 *malE*::Tn10	[65]

All strains listed as being from the Sandler lab are isogenic and are derivatives of JC13509. The genotype of JC13509 is *sulB103 lacMS286 φ80dIIlacBK1 argE3 hi-4 thi-1 xyl-5 mtl-1 rpsL31 tsx*. The *lacMS286 φ80dIIlacBK1* denote two partial non-overlapping deletions of the *lac* operon [68], [69].

The majority of mutant strains behaved like wild type in this screen. Known host mutants that do not support replication were easily identified (Figure S1, see plate #1), but no new candidates with this phenotype were observed. Several mutants displayed the phenotype of interest i.e. showed fewer or no colonies in the spots compared to wild type (Figure S1, see plate #1 and #9). The phenotype of these latter mutants was re-confirmed by infecting with Mu phage carrying a different antibiotic resistance marker (Mu::Amp) to ensure that the phenotype was independent of the antibiotic used for selection. The final set of 30 mutants displaying this phenotype is arranged in four panels below the control panel in Figure 2B. The mutants are classified broadly into genes known to affect DNA recombination/Repair, RNA-associated functions, ‘Other’ functions, and Mu receptor function. A more detailed description of gene function is listed in Table S1.

### Mu integration and replication in *E. coli* mutants defective in lysogen recovery

**Figure 3:**
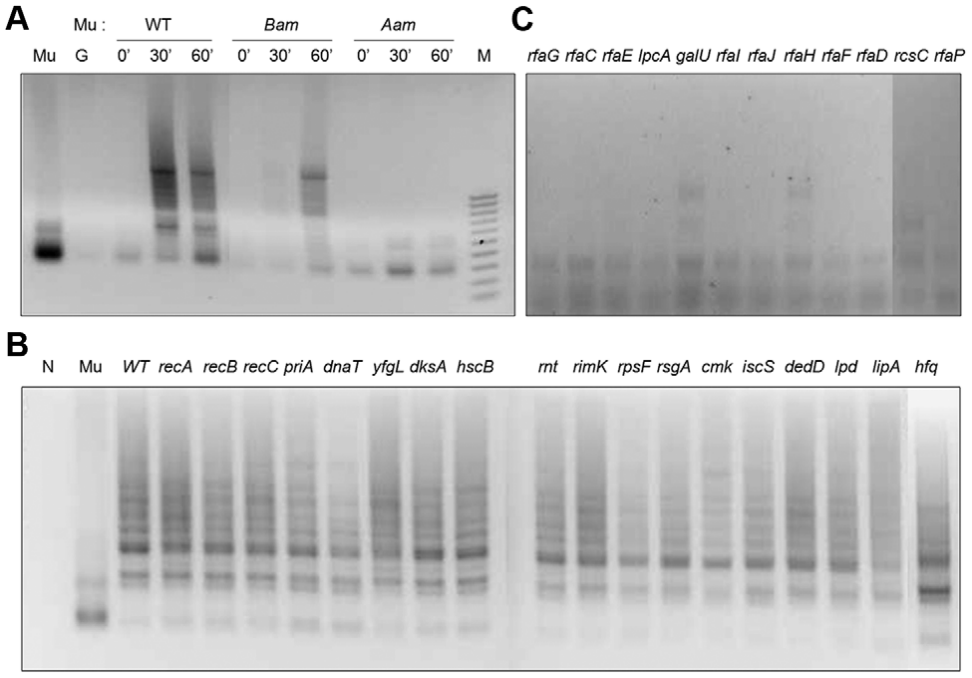
PCR assay for Mu integration in mutants defective in lysogen recovery. (A) Control PCR reactions monitoring integration at different time points after infection of wild type BW25113 with Mu::Cm, Mu::Cm(*Bam*1066) and Mu::Cm(*Aam*1093) phage. These phages can integrate-replicate, integrate but not replicate, or not integrate, respectively. (B) PCR results for wild type Mu::Cm integration 30 min after infection of mutants in the first three categories shown in Figure 2B. (C) As in (B) but with mutants in the Mu Receptor category. Control reactions with either no template (N), Mu, or genomic DNA templates from uninfected BW25113 host (G) are indicated, along with size markers (M). Reaction products were run on agarose gels and stained with ethidium bromide as described under Methods.

The poor yield of Cm^R^ colonies in the mutants shown in Figure 2B could be due to defects in Mu entry, integration, stable maintenance of lysogeny, or repair. To distinguish between some of these possibilities a PCR assay was first employed to test for Mu integration (Figure 3A). Two primers were chosen to amplify covalent junctions between the left end of Mu DNA and an arbitrarily chosen target gene *purH*. A PCR product is expected once the 3′ ends of Mu are joined to the target regardless of the fate of 5′ ends (see Figure 1). PCR products of different lengths are expected since Mu integration is essentially random [20], [21]. Using this method, a control experiment first followed the time course of wild type as well as mutant *Bam* and *Aam* Mu phage infections in the wild type strain. The particular *Bam* mutation used here (*Bam*1066) is reported to be fairly proficient in integration but defective in replicative transposition of Mu [22]. The *Aam* mutant (*Aam*1093) is defective in integration [23]. The integration patterns obtained during these infection experiments were consistent with the known transposition properties of these phages (Figure 3A).

Wild type Mu was used to infect the 30 mutants obtained in the initial screen for repair-defective mutants (Figure 2B). Mutants grouped under Recombination-Repair, RNA and Other categories all showed similar levels as well as patterns of integration compared to the wild type strain (Figure 3B). Quantitative PCR with a subset of these mutants (*priA, recA*) validated the results with normal PCR (Figure S2; we note that Southern blots used in earlier studies also showed similar levels of Mu integration in wild type and *priA* mutants [24]). Thus, these mutants were not defective in either Mu entry or integration. A majority of the mutants with defects in the LPS biosynthesis pathway, however, showed little or no integration (Figure 3C). This is likely due to a block in Mu entry, since the receptor for Mu is located within the LPS [25], [26].

To test if mutants that supported integration also supported Mu replication, cell lysis and phage production were monitored. Growth of the strains with and without Mu infection is shown in Figure S3. The LPS mutants in Figure 2B all grew as well as wild type; only a representative mutant *rfaF* is shown in Figure S3A. Neither this mutant, nor others in this category were susceptible to lysis by Mu infection (Figure S3B), supporting the conclusion that this group of mutants is defective in Mu entry. They were therefore not studied further. The remaining mutants showed varying degrees of growth impairment compared to wild type (Figure S3A). With the exception of *priA* and *dnaT*, which are essential for Mu replication [5], cell lysis and phage production were observed in all of the infected strains (Figure S3B). Thus, the majority of these mutants supported both Mu integration and replication. Their defect in yielding stable lysogens could therefore be due to an inability to maintain lysogeny or defects in repair of the insertions.

Defects in maintenance of the prophage state or lysogeny might be discerned by examining Mu plaque morphologies on these mutants. These would be expected to have a ‘clear’ rather than the ‘turbid’ phenotype observed for wild type Mu, which can be maintained in a lysogenic state. *dksA, hfq, rnt and rpsF* gave turbid plaque morphologies somewhat similar to the wild type strain, *dedD* was apparently clear, while the remaining mutants had clear centers and clear edges with turbid rings in-between (Figure S4). In the latter set of mutants with the mixed clear-turbid phenotype, it was difficult to ascertain whether the lysogeny-maintenance function might be affected.

### Keio mutant screen with replication-defective Mu

**Figure 4:**
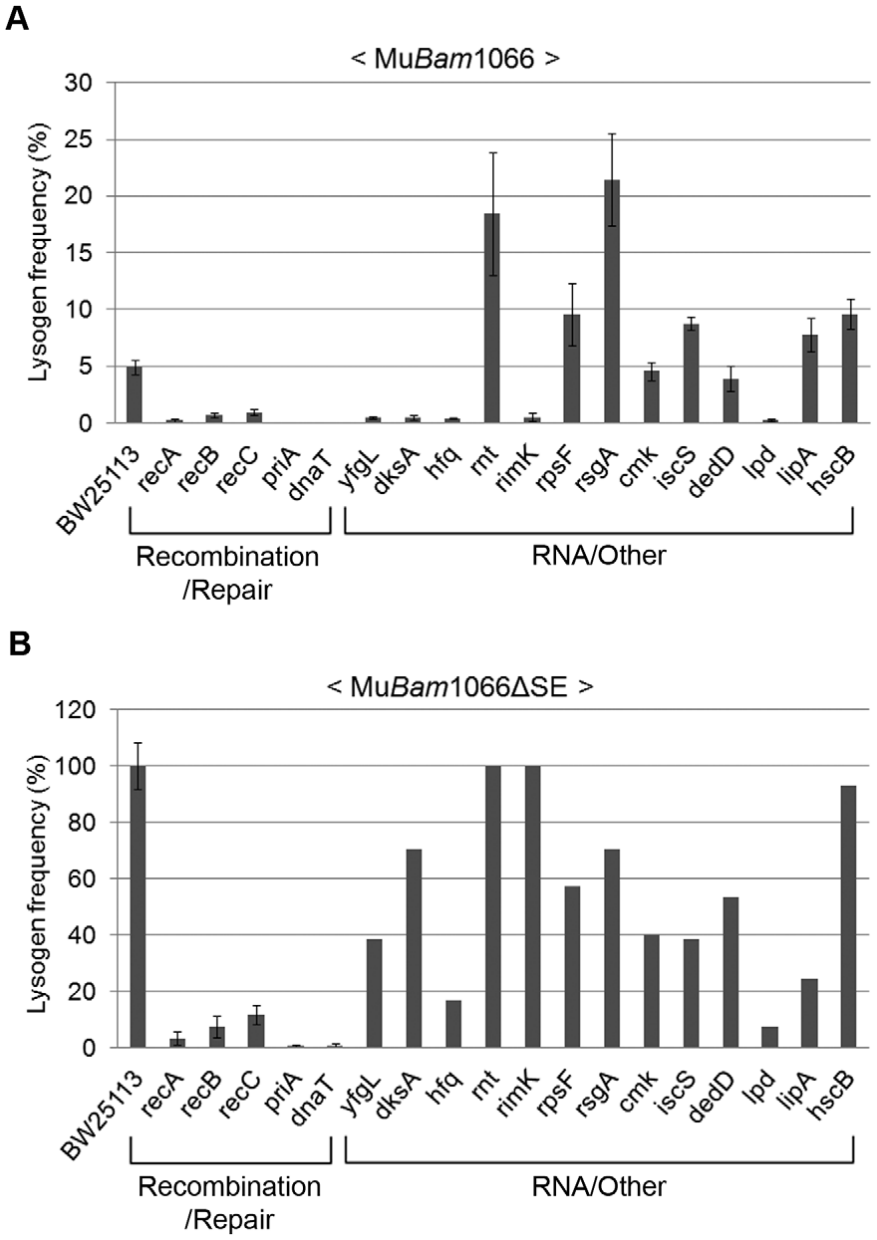
Mutant screen using replication-defective Mu. Lysogenization efficiencies (calculated as Cm^R^ cells/infected cells×100) of the mutant strains infected with either (A) Mu::Cm(*Bam*1066) or (B) Mu*Bam*1066ΔSE::Cm. Mutant categories as in Figure 2B. Error bars indicate standard deviation from the mean of triplicate data sets obtained from three independent colonies of the same strain. In (B), data for RNA/Other mutants are from a single colony/experiment. See Methods for details.

To eliminate scoring mutants as repair-defective because they were unable to maintain the lysogenic state and were therefore going lytic, we re-screened the Keio library with a Mu::Cm variant defective in replication. This phage carries the *Bam*1066 mutation, which allows integration but does not support replicative transposition (see Figure 3A; [22]). The same set of mutants was isolated in this screen as well. In the spot test results shown in Figure S5, it appears that some of the mutants have more Cm^R^ colonies than obtained with wild type phage (see Figure 2B). This is because a higher proportion of cells survive during infection with this phage due to absence of lytic growth. Lysogen recovery was therefore quantified as described under Methods (Figure 4A). Among mutants in the Recombination-Repair category, *priA* and *dnaT* mutants were the most severely affected in lysogen recovery (0.04%), followed by *recA* (0.2%), *recB* (0.7%) and *recC* (0.9%). Among mutants in the RNA and Other category, with the exception of *yfgL*, *dksA*, *hfq*, *rimK* and *lpd*, the remainder had lysogen frequencies similar to or even better than wild type.

A surprising aspect of the data shown in Figure 4A is that lysogen recovery in the wild type was only ∼5% with Mu*Bam* phage, and that cell viability after infection was only ∼20% (Figure S6A). Similar low cell viability was observed even after infection with integration-defective Mu*Aam* phage (Figure 3A and Figure S6A), which gave no Cm^R^ colonies. To test if this was due to expression of the cell killing function *kil* or to other function(s) specified by the unknown orfs in the SE (semi-essential) region [27], which is transcribed as part of a long early transcript that includes the *A* and *B* genes [2] (see Figure 2A), we deleted the SE region in the Mu*Bam* phage (see Methods). Indeed, infection with Mu*Bam*1066ΔSE::Cm phage improved both lysogen recovery and cell viability in the wild type to 100% (Figure 4B and Figure S6B, respectively). Under these conditions, all the mutants in the Recombination-Repair category still remained impaired (<15% of wild type) for lysogen recovery. In the RNA/Other category, *hfq*, *lpd* and *lipA* were also still substantially impaired (18–25% of wild type). Since *hfq* shows wild type plaque morphology (Figure S4) and since there is no obvious relationship of the known functions of these three genes to DNA repair, we will not consider them further here.

We conclude that a majority of the *E. coli* genes required for recovery of stable Mu insertions provide functions that apparently allow host survival in the presence of lethal phage functions specified by the SE region of Mu. The group of five genes that remain defective - *priA*, *dnaT*, *recA*, *recB* and *recC* – is significant in that this group is known to participate in recombinational repair. The isolation of this group of genes must be related to the repair of Mu insertions and not to repair of random double strand breaks generated upon Mu infection, because (1) they are dependent on Mu integration (i.e. infection with Mu*Aam*1093 phage does not significantly affect the viability of the *priA* and *recA* hosts as compared to wild type; Figure S6A), and (2) Mu-induced mutations are known to be tightly linked to Mu i.e. they are not random [28].

### Role of replication restart in the non-replicative pathway of Mu transposition

PriA and DnaT play a central role in the repair of nicks and gaps created by DNA damaging agents in *E. coli* by promoting replication restart after fork collapse, either with or without the involvement of recombination [29]. There are multiple pathways for replication restart that require PriA, PriB, PriC, DnaT and Rep [29]. These proteins identify the correct substrate, process it if necessary, and then aid DnaC in loading the replicative helicase DnaB during pre-primosome formation. PriA and DnaT are required for the two main pathways of ‘Restart’ where PriB and PriC have redundant roles. Thus *priA* and *dnaT* null mutants have extreme phenotypes whereas *priB* and *priC* null mutants have none. *dnaC809,820* is a *priC/rep*-independent suppressor that restores all known phenotypes of *priA* and *dnaT* null mutants [30]. During the lytic cycle of Mu growth, PriA restarts Mu replication without the involvement of homologous recombination ([24], [31] and Figure S3B). The data reported in Figure 2, Figure 3, and Figure 4 in this study show that PriA and DnaT are also required during the non-replicative event, along with a requirement for homologous recombination proteins.

**Figure 5:**
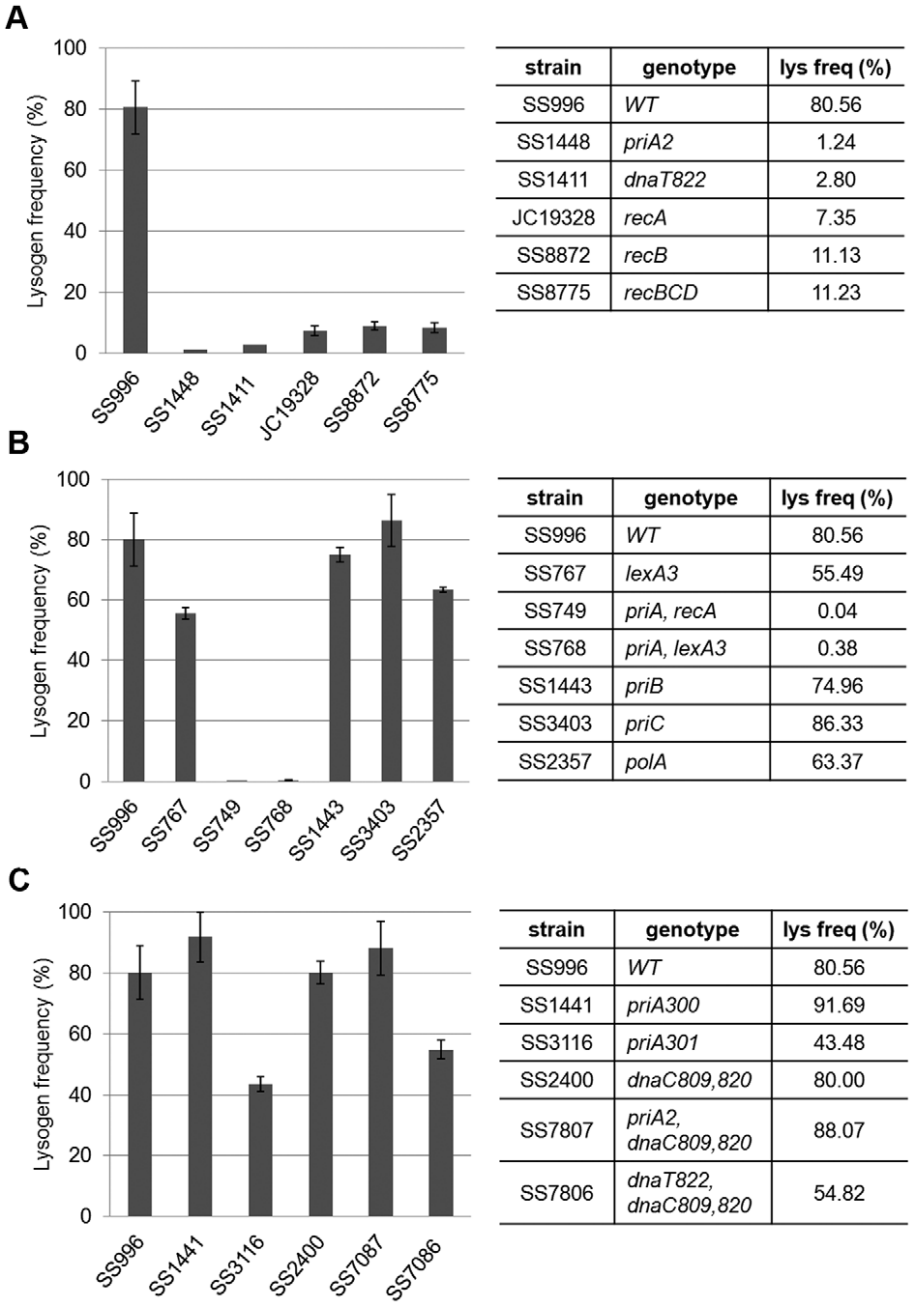
Behavior of various *priA*, *dnaT*, and *rec* alleles in a different strain background. Mu*Bam*1066ΔSE::Cm was used for infection of indicated strains to assess their role in recovery of Mu insertions. Other descriptions as in Figure 4.

To confirm the phenotype of *priA*, *dnaT*, and the *rec* genes and to dissect the role of PriA further, we tested these and several different mutant alleles of these genes in a different strain background. The *priA*, *dnaT*, *recA*, *recB* (and *recBCD*) mutants all showed defects in Mu lysogen recovery in this strain background as well (Figure 5A). *priA* and *dnaT* mutants show poor growth (Figure S3A and [32], [33], [34]) and many cells in the population have high levels of SOS expression [35]. SOS genes are normally kept silent by the repressor LexA, and activated only when LexA is cleaved by RecA in response to DNA damage [36]. SOS induction can be prevented by removing *recA* or by introducing a non-cleavable *lexA3* allele [37]. To test if SOS expression is responsible for the low recovery of Mu lysogens, we tested *priA lexA3* and *priA recA* double mutants; both mutants remained defective (Figure 5B). A *lexA3* mutant alone supported efficient recovery of Mu insertions, showing additionally that the SOS response is not required, but that the recombination function of RecA is needed. We note that *recA1*, a recombination-defective missense allele of *recA*, was not seen to affect recovery of Mu insertions in *Salmonella* [38], [39]. This allele can bind ssDNA *in vitro* [40], and perhaps has residual activity *in vivo* that allows it to function in Mu repair. We also note that several genes in the Keio collection were recently reported to be partially duplicated [41]. Of these, *priB* and *polA* are of interest to this study. These gene deletions as well as *priC* were therefore re-tested in the same strain background as the *priA* alleles. They were found to not affect Mu recovery (Figure 5B).

PriA has at least four types of activities: ATPase, helicase, the ability to load the replisome, and the ability to interact with other proteins. PriA300 (K230R) inactivates the ATPase and helicase activities, yet primosome assembly can occur both *in vivo* and *in vitro* [42], [43]. PriA301 (C479Y) mutates a residue in the cysteine-rich region of PriA thought to be important for protein-protein interactions and helicase activity [44]. Like *priA300, priA301* maintains wild-type growth and recombination proficiency [45], [46]. Lack of the helicase activity of PriA has been reported to impair Mu replication both *in vivo* and *in vitro* [31]. Using the helicase-defective strains *priA300* and *priA301*, we observed that the helicase and protein-protein interaction activities of PriA are largely dispensable (Figure 5C), indicating that it is the primosome activity of PriA that is essential for recovery of Mu insertions. This is further supported by the observation that combining *priA* and *dnaT* null mutations with *dnaC809,820* restores the ability of strains to recover lysogens (Figure 5C). Both *in vivo* and *in vitro* experiments have suggested that mutant DnaC proteins suppress the absence of PriA/DnaT complex by bypassing its role in helping DnaC to load DnaB/PolIII directly onto a recombinational intermediate [30], [47].

**Figure 6:**
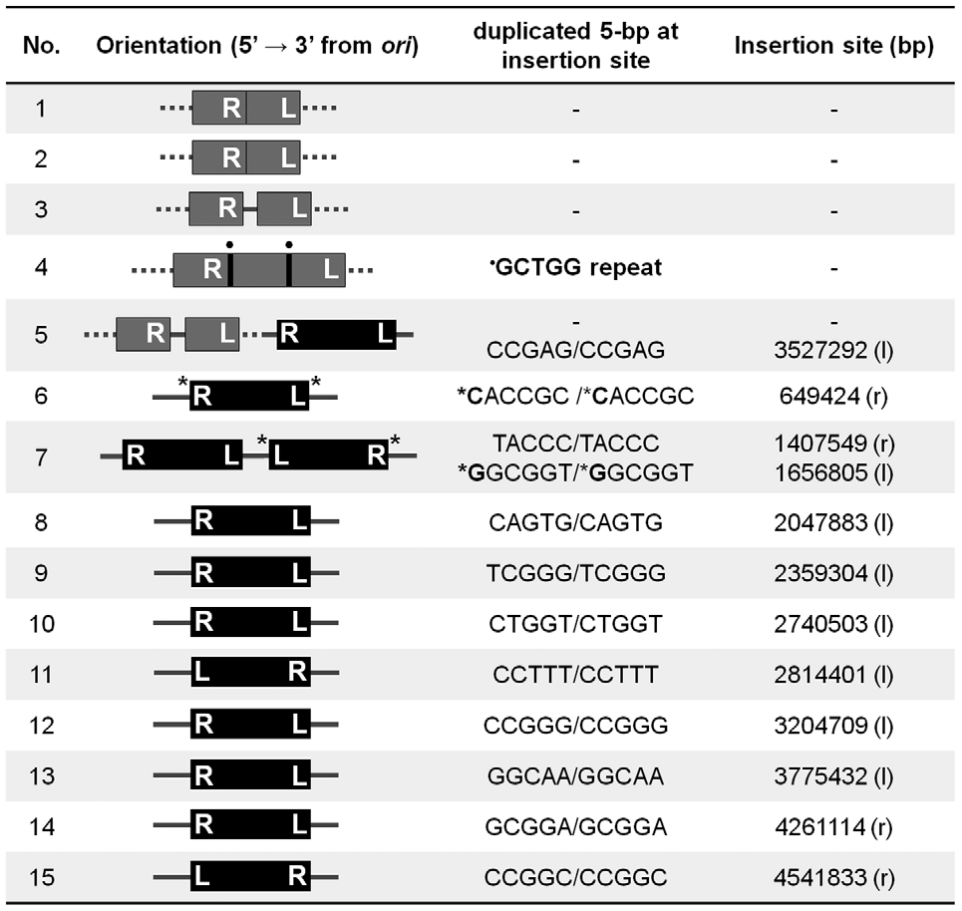
Sequence of Mu-host junctions at 15 insertions recovered in a *priA* mutant infected with Mu::Cm(*Bam*1066). See Methods for sequencing details. Orientation refers to clockwise positions of Mu from *oriC*, which is at ∼3.92 Mb; *ter* is at ∼1.59 Mb. The numbers in the Insertion site column refer to nucleotides on the *E. coli* genome. Black bars, intact Mu with L an R ends indicated; Gray bars, truncated/duplicated Mu with only one end identified; Dotted lines, undetermined host DNA sequence; • a repeated sequence; * insertion of nucleotides not found in the host DNA; l, r, position of insertions in the left and right replicores, respectively.

To confirm that all of these data point to a critical role for replication restart in repair of Mu insertions, we sequenced fifteen independent insertions which were recovered at a low frequency in the *priA* mutant (see Materials and Methods) (Figure 6). Of these, five insertions had rearranged the Mu-host junctions in various ways, and their precise location could not be determined. Two insertions had symmetrical additions (at both ends) of a nucleotide not found in the wild type host, likely due to repair by an error-prone polymerase, and one of these strains had two copies of Mu. Eight insertions had normal Mu-host junctions. We note that the sequencing strategy included cloning of Cm^R^ Mu DNA fragments, favoring recovery of R end fragments that had not been deleted or rearranged, and therefore underestimating the fraction of incorrectly repaired insertions. Overall, these results show that in the absence of PriA, Mu insertions are repaired inefficiently and often incorrectly by alternate pathways. Thus, PriA is indeed required for normal repair of Mu insertions.

## Discussion

Most transposable elements generate characteristic target site duplications flanking their insertion sites as a result of staggered cuts in the target initially made by the transposase [1]. For the large majority of known transposable elements whose transposition is not coupled to replication, it is not known how the single-stranded gaps left in the target after strand transfer are filled. For retroviruses and Line retroelements, double-strand break repair pathways (NHEJ, ATM, ATR) have been implicated [48], [49], [50], [51]. The present study finds that for Mu, in the non-replicative pathway, the gaps are repaired by the primary machinery for double-strand break repair in *E. coli* – the PriA primosome and homologous recombination proteins. This finding represents a radical change in thinking regarding Mu transposition in particular, and the transposition field in general. In the case of Mu, this is because one did not expect replicative functions to be involved in a transposition event that had been labeled ‘non-replicative’ in early studies. The original label was somewhat of a misnomer in that it described the replication status of Mu *prior* to integration [6], [7], [8]. However, discovery of flap DNA removal upon Mu integration [12], [13] meant that a second round of transposition could not occur until the gapped strand transfer intermediate was repaired. This event is therefore clearly different from the target-primed replication that immediately follows strand transfer during the replicative pathway. Early experiments that established the non-replicative transposition pathway found limited replication near the ends shortly after integration of infecting Mu, consistent with the idea of gap-filling repair [7]. We note that simple inserts generated using crude extracts and mini-Mu plasmids *in vitro* were also seen to have some replication associated with ends of Mu DNA, although it is not clear whether these simple insertion events are representative of the first integration event after Mu infection [52]. The identification of replication restart proteins in the present study suggests a new pathway for gap repair. These findings should spur a re-examination of similar assumptions made for other transposons that transpose by non-replicative mechanisms.

### Requirement for PriA in both replicative and non-replicative Mu transposition

There are three pathways for replication restart in *E. coli*: PriA–PriB, PriA–PriC, and PriC–Rep, which differ in their recognition of stalled forked structures [29]. PriA plays an essential role in initiation of replication on the forked DNA intermediates generated during the lytic phase of Mu growth, using either the PriA–PriB or PriA–PriC pathway, in addition to the proteins that are required for *E. coli* chromosomal replication [24], [31], [53], [54]. During Mu transposition, the transition from strand transfer to DNA replication can be divided into a number of discrete steps [3], [5]. MuA initially remains tightly bound to the Mu fork as a multi-subunit complex called transpososome. In a highly choreographed series of steps, host proteins dislodge this transpososome and assemble a replisome. In the first step of this transition, ClpX alters MuA subunit interactions to weaken interaction of the transpososome with DNA [55], [56], [57]. Next, as yet unidentified cellular factors called Mu Replication Factor α2 (MRF α2) displace the transpososome and exchange it with the translation initiation factor IF2-2 to produce a pre-replisome [58]. Finally, the helicase activity of PriA is required to displace IF2-2, remodeling the template to permit replisome assembly, which includes DnaT, DnaB, DnaC and the DNA polymerase III holoenzyme [5]. PriA has distinct replisome assembly and 3′ to 5′ helicase activities [29]. Helicase-defective PriA supports little or no Mu replication *in vitro*, and shows a partial defect in Mu replication *in vivo* [31]. These data indicate that PriA's replisome assembly activity is essential for initiation of Mu DNA replication and that the helicase activity also promotes this process. PriA is thought to bind to the lagging strand template at the fork and unwind it in a 3′ to 5′ direction, promoting loading of DnaB, thus coupling its replisome assembly and helicase activities.

The surprising requirement of PriA and DnaT in the non-replicative pathway of Mu transposition as reported in this study, suggests strongly that the 5 bp gaps generated upon Mu insertion are repaired by the replication restart machinery. This shared requirement for the PriA primosome in both pathways might imply that the PriA loading steps after strand transfer are similar in both. What apparently distinguishes the two pathways is non-requirement of the helicase activity of PriA, and requirement for homologous recombination proteins. We discuss two alternate models for recombinational gap repair below.

### Models for recombinational repair

**Figure 7:**
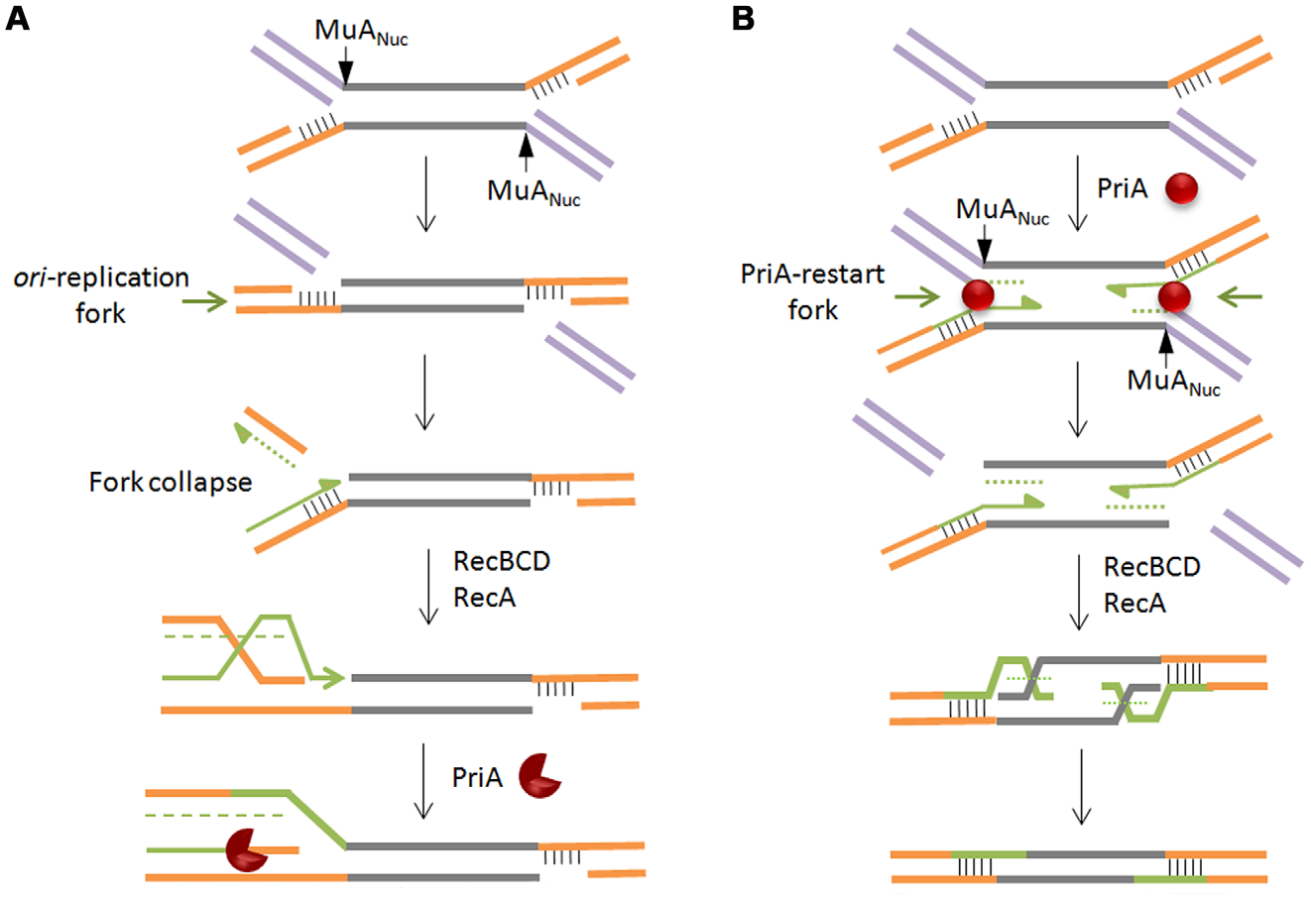
Models for recombinational repair of Mu insertions in the non-replicative pathway. Both models presented rely on repair of double strand breaks by homologous recombination and replication restart proteins, but differ in the location of the break and the order of the recombination/restart-replication events that follow. In (A), the break is on the chromosomal DNA flanking the Mu insertion. Here, homologous recombination is followed by restart replication. In (B), the break is on the Mu lagging strand. Here, restart replication precedes homologous recombination. Alternate shapes for PriA denote uni- or bi-directional replication. See text for details.

Nicks and gaps in DNA are normally repaired when their encounter with a traveling replication fork converts them into a double strand break, collapsing the fork [36]. The broken end serves as an entry point for RecBCD, generating single strands for RecA binding, followed by invasion of the intact sister chromosome, thus reconstituting a forked structure for restarting replication via the PriA primosome [59], [60]. In such a scenario for Mu repair, an *oriC*-initated fork will cause a double strand break when, arriving at the site of a Mu insertion, it encounters the flanking gap (Figure 7A). The double-strand break will be on the chromosomal DNA flanking the Mu insertion, which is expected to be processed by RecBCD, followed by restoration of the fork by recombination, and restart of replication by the primosome. Two considerations make this scenario unappealing. First, Mu does not insert near replication forks [61], so the unrepaired intermediate would be potentially vulnerable to degradation while it waits for the *oriC*-initiated fork to arrive. Second, the passing fork would encounter only one of the two gaps at each Mu end that need repair, so the entire Mu would have to be replicated, generating a second double strand break at the distal Mu end, reiterating RecA-mediated invasion and primosome assembly before repair of the second gap can be completed. A parsimonious alternative model takes advantage of the PriA replisome already present at the forked strand transfer joints at both Mu ends, recruited there in the normal course of transpososome disassembly (see Figure 1). In this model, the initial steps of PriA recruitment and replication are common to both the repair and replication pathways (Figure 7B). The pathways differ in the flap cleavage step, which ensues concomitant with replication restart, leaving double-strand breaks on the Mu lagging strand. These breaks allow RecBCD entry, creating single-stranded 5′ Mu ends on which RecA polymerizes [62]. Although 3′ end strand invasion is generally preferred with purified RecA, 5′ ends can be used for strand exchange *in vitro* [63], and *in vivo* recombination data also fit models that invoke 5′ strand invasion [64]. The Holliday junction so created can then be resolved by Ruv proteins or endonucleases. This model reverses the steps normally associated with recombinational repair, with replication preceding recombination. According to this model, there will be limited replication near the two Mu ends in this largely non-replicative event.

What signals flap cleavage in one pathway and not in the other? We speculate that the MuN protein, which normally protects the ends of infecting Mu DNA from degradation, dissociates from the ends, perhaps upon interaction with the transpososome assembled on the strand transfer complex. This allows RecBC to enter and peel away the 3′ strand of the flap, engaging and activating MuA_Nuc_ on the 5′ strand.

### Summary

This is the first report of specific host processes involved in repair of transposon insertions in bacteria. We find that the PriA primosome and homologous recombination proteins, which are essential for repair of double-strand breaks in *E. coli*, play a critical role in the repair of Mu insertions. We favor a model for recombinational repair in which PriA restart of Mu replication is followed by RecA-mediated resolution of double-strand breaks on the Mu lagging strands created by the flap endonuclease activity of the transposase. Given that the predominant route taken by Mu upon infection is to enter lytic growth, it is plausible that Mu first co-opted the PriA system for replication, and later used it for repair. It will be interesting to see whether other transposons use these same processes for repair of their insertions.

## Materials and Methods

### Strain construction

All strains used in this work are derivatives of *E. coli* K-12 and are listed in Table 1 [13], [19], [22], [23], [34], [35], [65], [66]. The Keio Collection (single-gene knockout library of 3,985 nonessential genes in *E. coli*) was obtained from the National BioResource Project, Japan. The wild type strain in this collection is BW25113. *E. coli* Mu lysogen strains BU1717 or MH3491 were used to construct strains SJ17 – SJ19 (Table 1), where a ∼1 kb *cat* cassette was inserted downstream of the invertible G-segment on the Mu genome at nt 35,040, before *gin*, by the method of Datsenko and Wanner [67]. The SE deletion was similarly constructed; it removes nt 4,319–7,954 from the Mu genome, substituting the *cat* cassette in its place. All Mu phages used in this study carry the temperature-sensitive *ts*62 allele of the lysogenic repressor gene *c*. Primers used in this study are listed in Table S2.

### High-throughput screening of the Keio library

Cultures from the Keio collection stocked in 96-well plates were inoculated into new sterilized 96-well plates with 0.2 ml of Luria broth (LB) by using the 12-multichannel pipette (Biohit). They were incubated at 37°C overnight without shaking. 4 µl of saturated overnight cultures were transferred to 0.2 ml of fresh LB media supplemented with 2.5 mM CaCl_2_ and 5 mM MgSO_4_ in 96-well plates and incubated at 37°C until OD_600_ reached around 0.5, measured directly in the plates by DTX880 microplate reader (Beckman). Mu phage was added to the cultures at a multiplicity of infection (moi) of 5, mixed briefly, and incubated at 30°C for 1 hr. 4 µl of infected cultures were spotted on slab agar plates having dimensions similar to the 96-well plates and containing 25 µg/ml chloramphenicol; plates were incubated overnight at 30°C.

### PCR–based assay for Mu DNA integration

50 µl overnight cultures were transferred to 5 ml of fresh LB media supplemented with 2.5 mM CaCl_2_ and 5 mM MgSO_4_ and grown to 0.5 at an OD_600_. Phage were added to the cultures at 5 moi and incubated at 30°C for 30 min. Infected cells were harvested and the total DNA were isolated by Wizard Genomic DNA purification kit (Promega). PCR was conducted with 50 ng DNA as a template, 10 pmol primers, 1× *Go Taq* master mix (Promega), and distilled water up to 50 µl. Primers were designed to anneal to the left end of Mu DNA and the *purH* gene of *E. coli*. PCR conditions were: 94°C for 2 min, 30 cycles of - 94°C for 30 sec, 50°C for 30 sec, 72°C for 2 min 30 sec - and a final extension at 72°C for 2 min. PCR amplification primers used in this study are listed in Table S2. The reaction products were electrophoresed on 1% agarose gels and visualized by staining with ethidium bromide.

### Quantitative real-time PCR analysis

This method measures DNA amounts based on the fluorescence signal from SYBR-bound DNA. PCR reactions were conducted with the same templates and primers as used for normal PCR, with the additional inclusion of 1× Power SYBR Green PCR Master Mix (Applied Biosystems), and distilled water up to 25 µl. The PCR program in the 7900HT sequence detector (Applied Biosystems) was as follows: 95°C for 10 min, followed by several cycles of - 95°C for 30 sec, 50°C for 30 sec, 72°C for 2 min 30 sec. Cumulative fluorescence was measured at the beginning of the exponential phase of the PCR reaction to determine the fractional cycle number (*C*_T_). The level of integrated Mu DNA was normalized to a chromosomal locus *dnaC*, amplified with appropriate primers listed in Table S2.

### Growth curves

100 µl of saturated overnight cultures were transferred to 10 ml of fresh LB media and incubated at 37°C until OD_600_ reached around 0.5 for all cultures. From then on, growth was monitored by measuring OD_600_ at various times for 2 hr. A similar procedure was followed for obtaining lytic growth curves, except that the LB media was supplemented with 2.5 mM CaCl_2_ and 5 mM MgSO_4_. At OD_600_ of around 0.5, Mu phage was added at 5 moi, mixed briefly, and incubated at 37°C for 3 hr until most cultures were completely lysed. In all cases where *priA2::kan*, *dnaT822* (without *dnaC* mutations) or *polA*::*kan* strains were used, these were grown overnight in minimal media, followed by dilution into fresh LB media, and then allowed to grow into log phase before infection with the different Mu phages.

### Phage

These were prepared by induction of the prophage strains by thermal inactivation of the temperature-sensitive (*ts*) phage repressor *c*, and concentrated by CsCl gradient centrifugation as described [12]. For strains BU1717 (Mu*Bam*1066), SJ17 (Mu::Cm(*Bam*1066)) and SJ18 (Mu*Bam*1066ΔSE::Cm), the prophages were induced in the presence of pJG4 (c-myc MuB expressed from pET28(a) without IPTG induction) to supplement MuB protein. Typical phage titers after concentration were ∼10^11^ pfu (plaque forming units) for wild type Mu, and ∼10^10^ pfu for the *Bam* or *Bam*ΔSE phage. Phage titers for wild type Mu with and without the *cat* insertion were similar, showing that the insertion did not affect phage yields.

### Lysogenization/survival frequency

Cultures were infected with Mu::Cm(*Bam*1066), Mu*Bam*1066ΔSE::Cm or Mu::Cm(*Aam*1093) phage as described under ‘PCR-based assay for Mu integration’. Before and after infection, appropriate dilutions of cells in LB media were spread onto agar plates with or without 25 µg/ml chloramphenicol to obtain cell counts for input cells, survivors after infection, and lysogens. Plates were incubated at 30°C overnight, and colonies were counted the next day. Lysogenization efficiency was calculated as Cm^R^ cells/input cells×100, and survival efficiency was calculated as survivors (on non-antibiotic plate)/input cells×100.

### Sequencing Mu insertion sites in the *priA* mutant

*priA* lysogens were selected as Cm^R^ colonies after infection with Mu::Cm(*Bam*1066) phage. After overnight culture into LB media, chromosomal DNA was isolated by Wizard Genomic DNA purification kit and digested by restriction enzyme *BamH*I and *Pst*I. Digested DNA fragments were purified and ligated with similarly digested pUC19 plasmid. Cm^R^ transformants were isolated and digested by *BamH*I and *Pst*I to ascertain that the insert size was larger than 4 kb, so that it included DNA flanking the insertion. R1 primer (Table S2) was annealed to Mu DNA right end to obtain sequence of the flanking DNA. Based on this sequence, appropriate primers were used to PCR-amplify DNA flanking the left end of the insertion using the L1 primer. DNA sequencing was performed at our core sequencing facility.

### Plaque morphology

10 µl of an appropriate dilution of phage suspension were mixed with 100 µl of host cells grown to 0.5–0.6 at OD_600_ in LB including 2.5 mM CaCl_2_ and 5 mM MgSO_4_. The mixture was added to 3 ml of 0.3% molten soft agar at 42°C, and poured on top of an LB agar plate containing 2.5 mM CaCl_2_ and 5 mM MgSO_4_. Plates were incubated overnight at 37°C.

## Supporting Information

Figure S1 Initial results of spotting Mu-infected cultures derived from Keio plates #1 and #9 on LB Cm plates. X marks empty spots with no bacteria.
(TIF)

Figure S2 Quantitation of Mu DNA integration in wild type, *priA* and *recA* mutant strains by real-time PCR analysis. Genomic DNA isolated from the indicated Mu-infected strains was used in real-time quantitative PCR reactions to quantify Mu integration as described in Methods. *C*_T_ is the fractional cycle number at the beginning of the exponential reaction phase where the fluorescence passes a threshold (T) at which the fluorescence signal is first detected. *C*_T_ values are inversely proportional to the amount of amplified DNA. Δ*C*_T_ = Mu *C*_T_ – *dnaC C*_T_. *dnaC* is used as a control for as a single-copy chromosomal gene. The data are an average of three technical repeats.
(TIF)

Figure S3 Mu replication in mutants defective in lysogen recovery. (A) Growth curves of mutants, color-coded to indicate slow (red), medium (purple) or near-wild type (blue) growth patterns. (B) Lysis profiles of mutants after infection with wild type Mu, color-coded to indicate similarity to wild type (blue), slightly delayed from wild type (red), growth delay but no lysis (green), and no lysis (black). All strains were grown to OD_600_ of ∼0.5 prior before infection with Mu::Cm. Phage production in the lysed cultures was monitored by determining pfu.
(TIF)

Figure S4 Plaque morphologies of wild type Mu::Cm on Keio mutant strains defective in lysogen recovery. See Figure 2B.
(TIF)

Figure S5 Keio mutant screen using Mu::Cm(*Bam*1066). Final set of mutants obtained are shown. Spot tests and mutant categories are as in Figure 2B, except that strains in the control panel are all derived from BW25113. *himA* (*ihfA*) and *himD* (*ihfB*) code for the two subunits of IHF, which is essential for the Mu replicative pathway.
(TIF)

Figure S6 Survival efficiency of mutant strains infected with (A) Mu::Cm(*Bam*1066) and Mu::Cm(*Aam*1093) or (B) Mu*Bam*1066ΔSE::Cm phage. Survival efficiency is calculated as cells recovered after infection on no-antibiotic plates/infected cells×100. See Methods and Figure 4 legend for other details.
(TIF)

Table S1 Description of mutants defective in Mu lysogen recovery. ID numbers and associated gene descriptions are from the Keio web site www.ecolicommunity.org/genobase.
(RTF)

Table S2 Oligonucleotide primers used for PCR amplification.
(RTF)
