# Supplementary figures and images for: Mu Insertions Are Repaired by the Double-Strand Break Repair Pathway of Escherichia coli
Source: PLoS Genet. 2012 Apr 12;8(4):e1002642. doi: 10.1371/journal.pgen.1002642 (PMC3325207; doi:10.1371/journal.pgen.1002642)

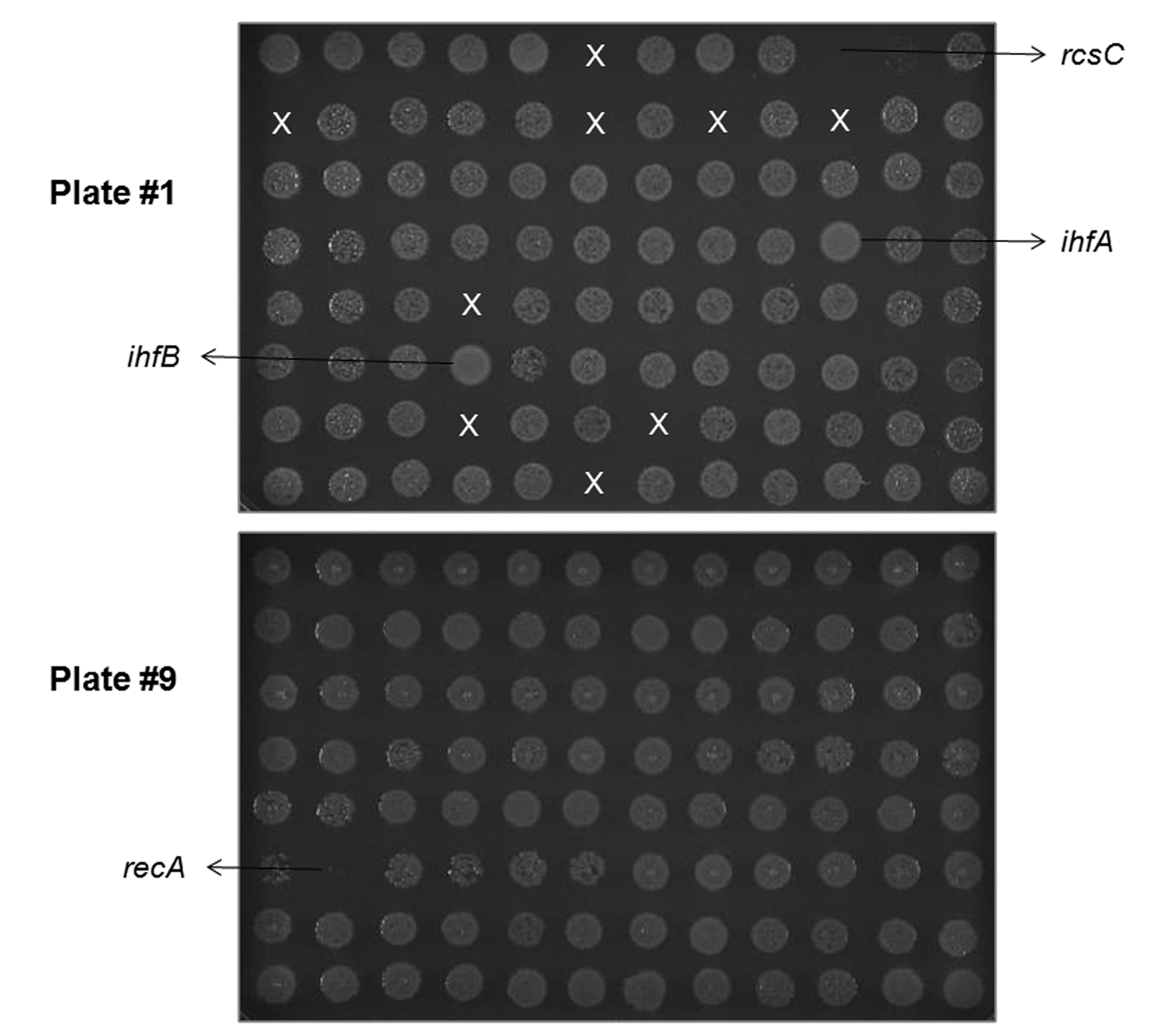

Supplement: Figure S1 — Initial results of spotting Mu-infected cultures derived from Keio plates #1 and #9 on LB Cm plates. X marks empty spots with no bacteria. (TIF) [file pgen.1002642.s001.tif]

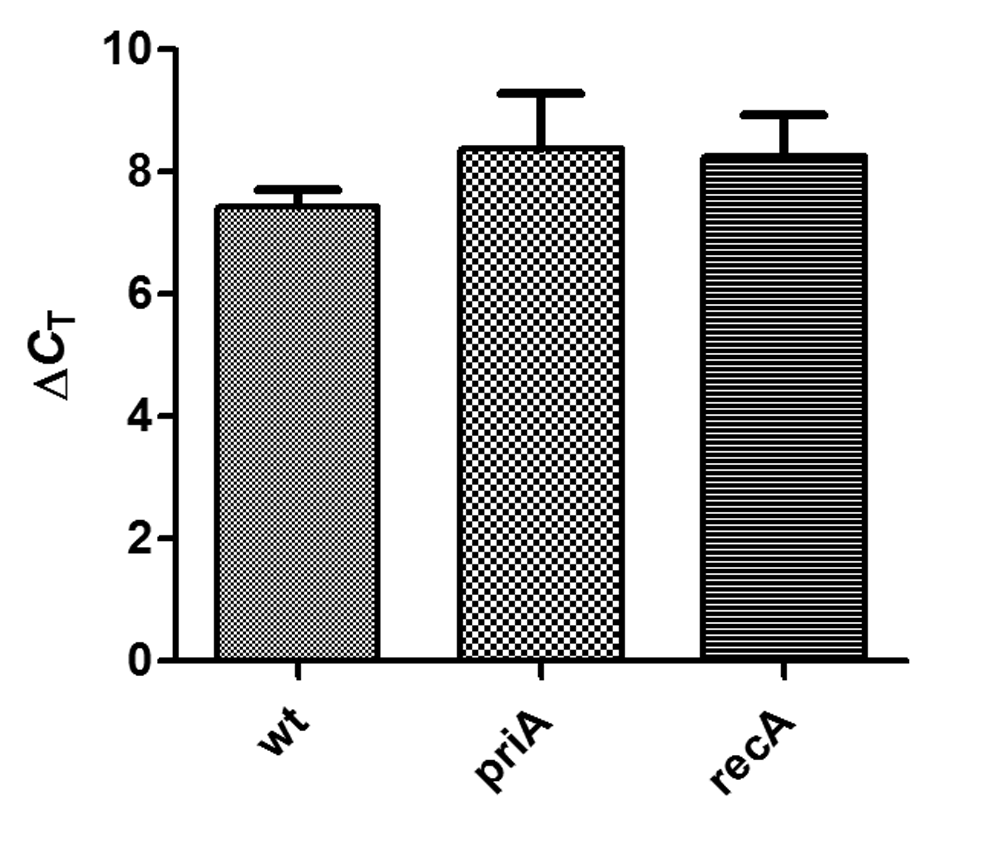

Supplement: Figure S2 — Quantitation of Mu DNA integration in wild type, priA and recA mutant strains by real-time PCR analysis. Genomic DNA isolated from the indicated Mu-infected strains was used in real-time quantitative PCR reactions to quantify Mu integration as described in Methods. C T is the fractional cycle number at the beginning of the exponential reaction phase where the fluorescence passes a threshold (T) at which the fluorescence signal is first detected. C T values are inversely proportional to the amount of amplified DNA. ΔC T = Mu C T – dnaC C T. dnaC is used as a control for as a single-copy chromosomal gene. The data are an average of three technical repeats. (TIF) [file pgen.1002642.s002.tif]

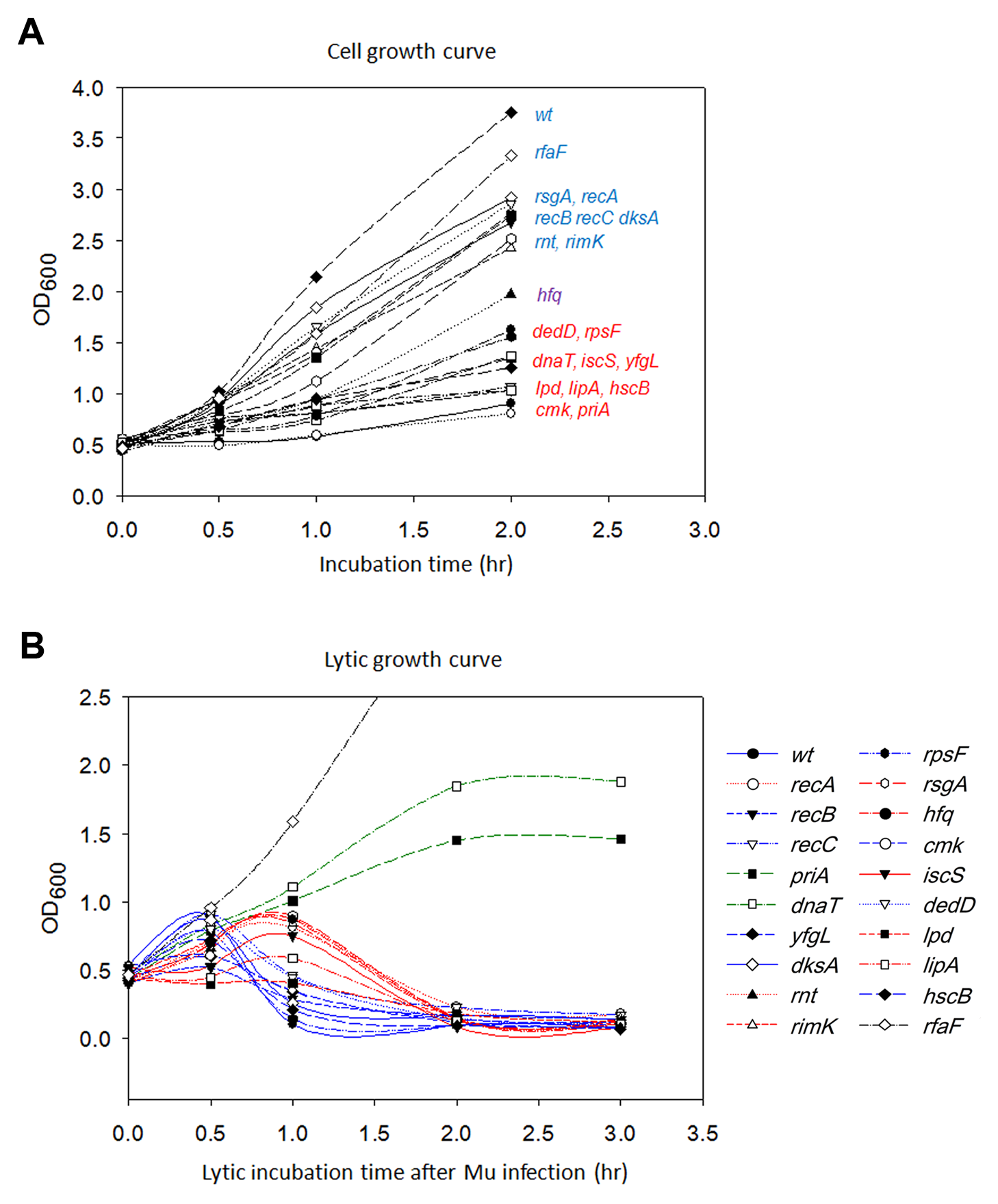

Supplement: Figure S3 — Mu replication in mutants defective in lysogen recovery. (A) Growth curves of mutants, color-coded to indicate slow (red), medium (purple) or near-wild type (blue) growth patterns. (B) Lysis profiles of mutants after infection with wild type Mu, color-coded to indicate similarity to wild type (blue), slightly delayed from wild type (red), growth delay but no lysis (green), and no lysis (black). All strains were grown to OD600 of ∼0.5 prior before infection with Mu::Cm. Phage production in the lysed cultures was monitored by determining pfu. (TIF) [file pgen.1002642.s003.tif]

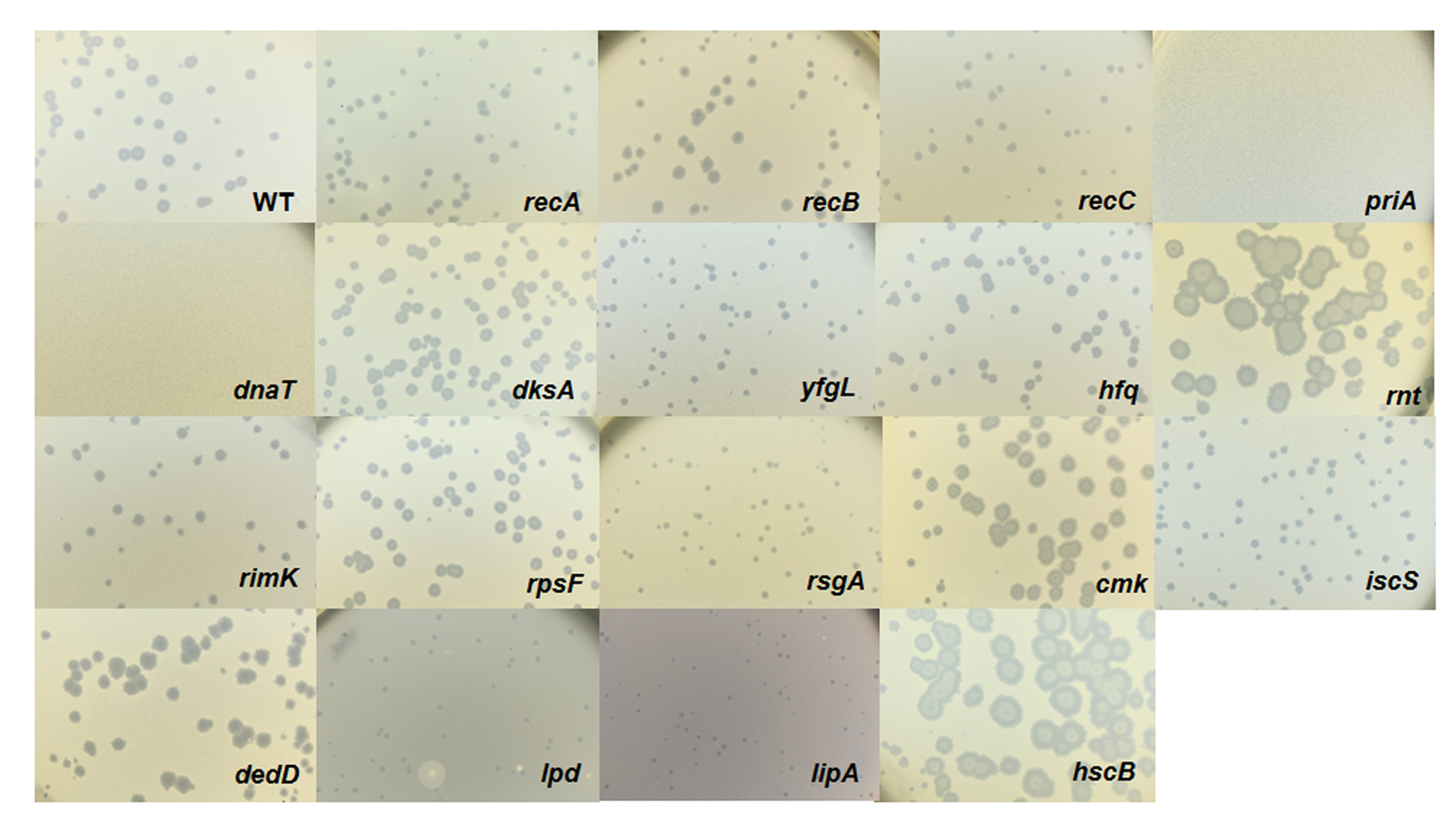

Supplement: Figure S4 — Plaque morphologies of wild type Mu::Cm on Keio mutant strains defective in lysogen recovery. See Figure 2B. (TIF) [file pgen.1002642.s004.tif]

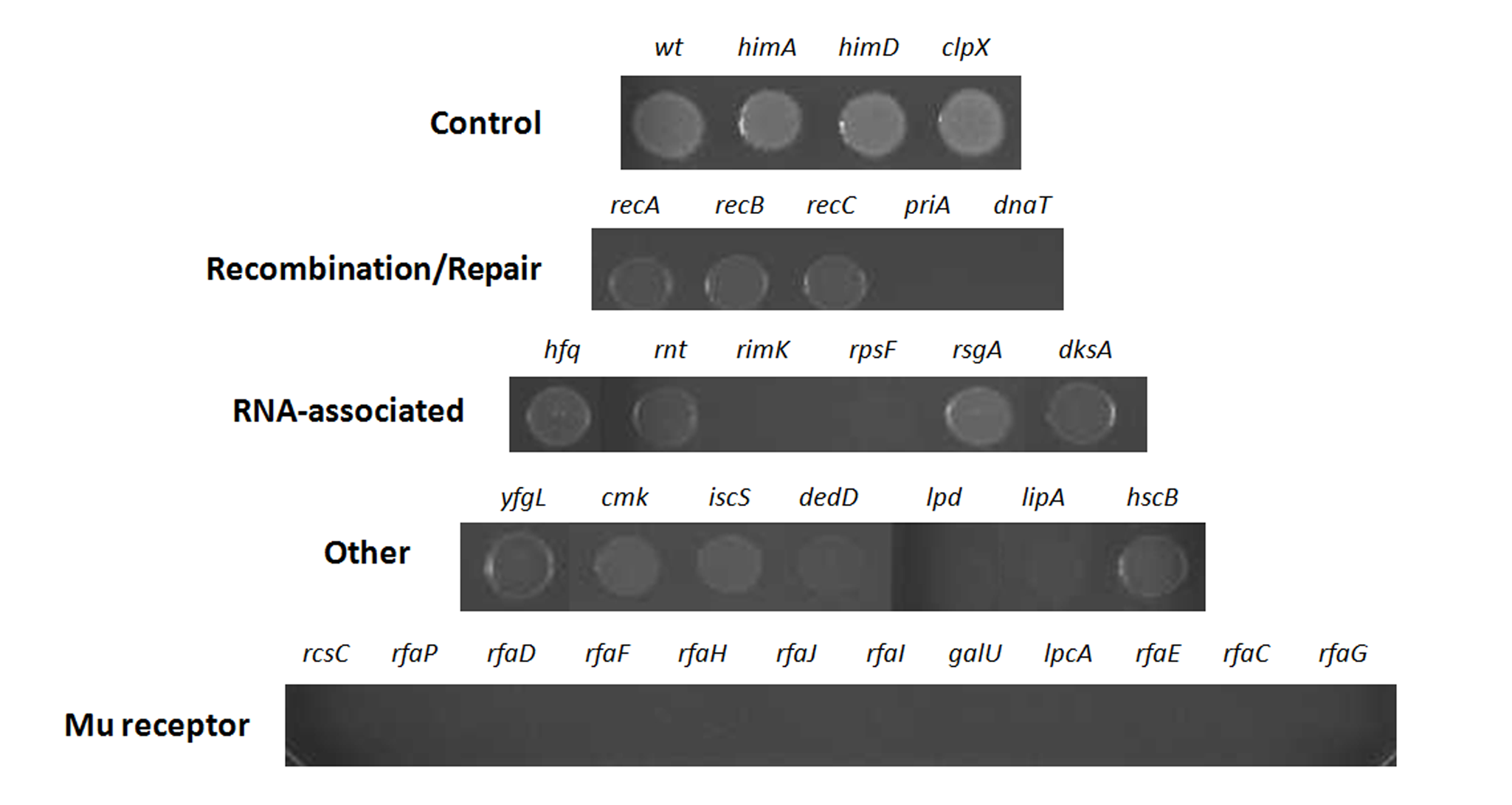

Supplement: Figure S5 — Keio mutant screen using Mu::Cm(Bam1066). Final set of mutants obtained are shown. Spot tests and mutant categories are as in Figure 2B, except that strains in the control panel are all derived from BW25113. himA (ihfA) and himD (ihfB) code for the two subunits of IHF, which is essential for the Mu replicative pathway. (TIF) [file pgen.1002642.s005.tif]

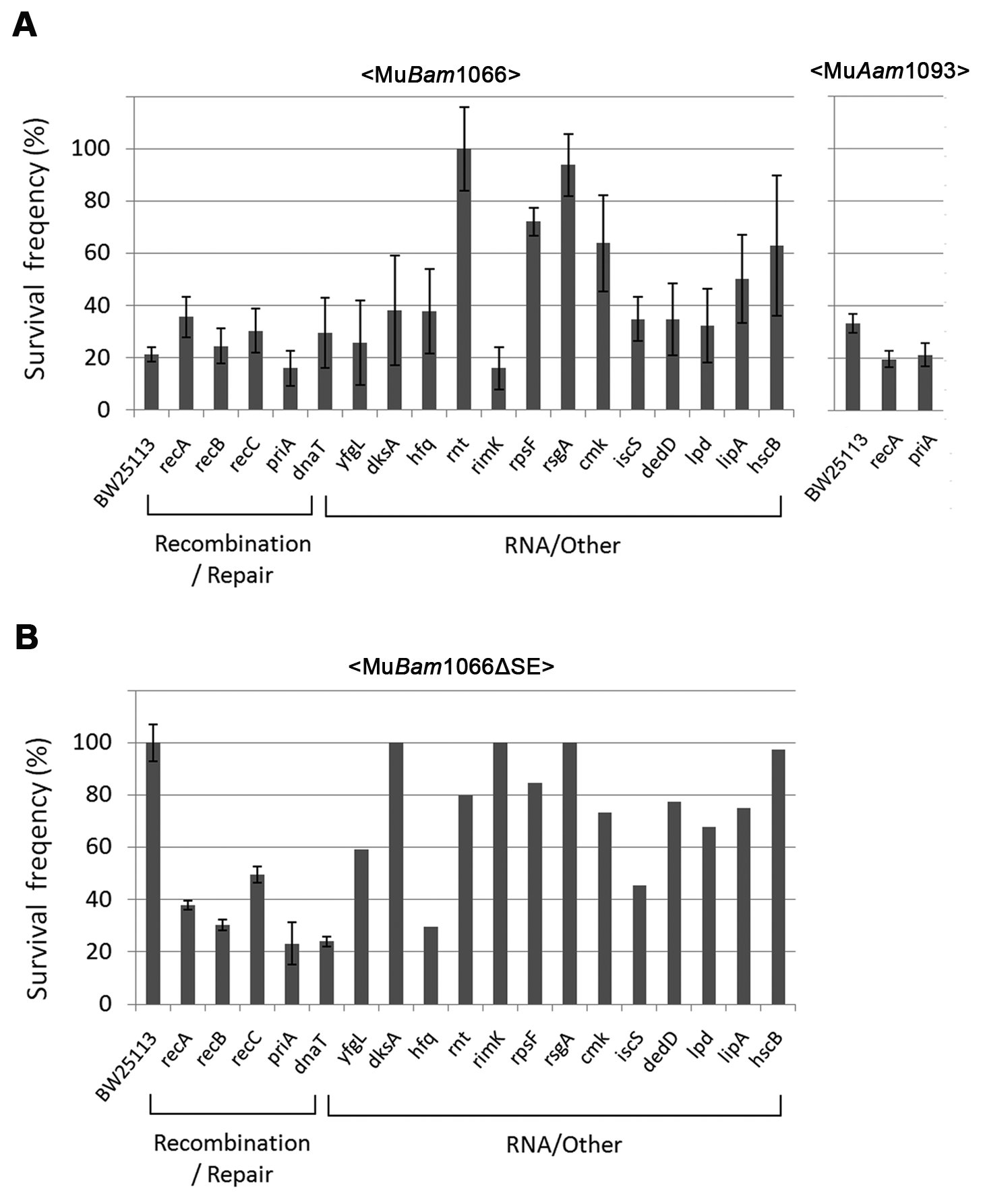

Supplement: Figure S6 — Survival efficiency of mutant strains infected with (A) Mu::Cm(Bam1066) and Mu::Cm(Aam1093) or (B) MuBam1066ΔSE::Cm phage. Survival efficiency is calculated as cells recovered after infection on no-antibiotic plates/infected cells×100. See Methods and Figure 4 legend for other details. (TIF) [file pgen.1002642.s006.tif]
