# Supplementary material for: Mu Insertions Are Repaired by the Double-Strand Break Repair Pathway of Escherichia coli
Source: PLoS Genet. 2012 Apr 12;8(4):e1002642. doi: 10.1371/journal.pgen.1002642 (PMC3325207; doi:10.1371/journal.pgen.1002642)
Supplement: Table S1 — Description of mutants defective in Mu lysogen recovery. ID numbers and associated gene descriptions are from the Keio web site www.ecolicommunity.org/genobase. (RTF) [file pgen.1002642.s007.rtf]

A. Defect in lysogen recovery
No.	ID (Keio)	Gene	Function	
GROUP 1. Repair/Recombination associated proteins	
1	2669	recA 	DNA strand exchange and recombination protein with protease and nuclease activity	
2	2788	recB	Exonuclease V (RecBCD complex), beta subunit	
3	2790	recC 	Exonuclease V (RecBCD complex), gamma chain	
4	3906	priA 	Primosome factor n' (replication factor Y)	
5	4326	dnaT 	DNA biosynthesis protein (primosomal protein I)	
GROUP 2. Ribosomal RNA associated proteins 	
6	141	dksA 	Transcriptional regulator of rRNA transcription, DnaK suppressor protein	
7	4130 	hfq 	RNA-binding protein that affects many cellular processes 	
8	1644	rnt 	Ribonuclease T (RNase T) 	
9	836	rimK 	Ribosomal protein L6 modification protein	
10	4158	rpsF 	30S ribosomal subunit protein S6	
11	4122	rsgA	Ribosome small subunit-dependent GTPase A	
GROUP 3. No category (Other) 	
12	2496	yfgL 	protein assembly complex, lipoprotein component	
13	2511	hscB 	Hsc20 co-chaperone that acts with Hsc66 in IscU iron-sulfur cluster assembly 	
14	893	cmk 	Cytidylate kinase 	
15	2514	iscS 	Cysteine desulfurase (tRNA sulfurtransferase), PLP-dependent 	
16	112 	lpd 	Lipoamide dehydrogenase, NADH-dependent 	
17	623 	lipA 	Lipid synthesis, iron-sulfur protein	
18	5378	dedD	Membrane-anchored periplasmic protein involved in septation 	


B. Defect in Mu DNA entry
No.	ID (Keio)	Gene	Function	
1	1224	galU	glucose-1-phosphate uridylyltransferase	
2	212	lpcA	D-sedoheptulose 7-phosphate isomerase	
3	5917	rcsC	hybrid sensory kinase in two-component regulatory system with RcsB and YojN	
4	3596	rfaC	ADP-heptose:LPS heptosyl transferase I	
5	3594	rfaD	ADP-L-glycero-D-mannoheptose-6-epimerase, NAD(P)-binding	
6	3024	rfaE	fused heptose 7-phosphate kinase/heptose 1-phosphate adenyltransferase	
7	3595	rfaF	ADP-heptose:LPS heptosyltransferase II	
8	3606	rfaG	glucosyltransferase I	
9	3818	rfaH	DNA-binding transcriptional antiterminator	
10	3602	rfaI	UDP-D-galactose:(glucosyl)lipopolysaccharide-alpha-1,3-D-galactosyltransferase	
11	3601	rfaJ	UDP-D-glucose:(galactosyl)lipopolysaccharide glucosyltransferase	
12	3605	rfaP	kinase that phosphorylates core heptose of lipopolysaccharide	
