# Supplementary material for: Mu Insertions Are Repaired by the Double-Strand Break Repair Pathway of Escherichia coli
Source: PLoS Genet. 2012 Apr 12;8(4):e1002642. doi: 10.1371/journal.pgen.1002642 (PMC3325207; doi:10.1371/journal.pgen.1002642)
Supplement: Table S2 — Oligonucleotide primers used for PCR amplification. (RTF) [file pgen.1002642.s008.rtf]

Primer	Sequence (5' -> 3')	
Insertion of cat into 35040 nt of Mu	
Owy185 (F) 	gccagaagcctgatttaccgtttcctgtaaaccgaggttttggataatggggatccgtgtaggctggagctgcttc	
Owy186 (R)	ctggatctcctgtttaaaagcgccaatcatgccatgcgtgccaaaatcgaggatcccatatgaatatcctcctta	
Substituting SE region with cat	
Owy061 (F)	atatttcaacgctgctgcgtaattaagaaggagaagaaattatgatggtgtaggctggagctgcttcg	
Owy062 (R)	cgccgcgaaaaactgcaactgtcaaagatcatggaagacattatcacatatgaatatcctcctta	
Monitoring integration of Mu DNA into host chromosome	
Mu_L1 	gcttggttgttatcggttttgaacg	
purH_R 	cttcgacaaactgacgagaa	
dnaC_F	ttgtgtcgcagtataccgc	
dnaC_R	gaacttgacagccaaattcc	
Sequencing Mu insertion site	
Mu_R1 	gctacatcagattcctgaacaaacg	
Mu_L1 	gcttggttgttatcggttttgaacg	
